# Solid state pelletizing for the synthesis of new Bi-doped strontium molybdate and its development as a photocatalytic precursor for Rhodamine B degradation

**DOI:** 10.1016/j.mex.2023.102258

**Published:** 2023-06-15

**Authors:** Jorge Acosta-Vergara, Ricardo A. Torres- Palma, Yenny Ávila- Torres

**Affiliations:** a Department of Macromolecular Compounds, Faculty of Chemistry, Lomonosov Moscow State University MSU, Moscow 1, GSP-1, 1-3 Leninskiye Gory, Moscow 119991, Russia; b Grupo de Investigación en Remediación Ambiental y Biocatálisis (GIRAB), Instituto de Química, Facultad de Ciencias Exactas y Naturales, Universidad de Antioquia UdeA, Calle 70 No. 52-21, Medellín, Colombia

**Keywords:** Synthesis, Strontium molybdates, Bismuth, Band-gap, Estimation photocatalytic, *Solid state reactions of new Bi-doped strontium molybdate synthesized as photocatalytic precursors for Rhodamine B degradation*

## Abstract

At present, climate change, urbanization and globalization are the main factors that affect water quality, the primary vehicle for the translocation and permanence of emerging pollutants, resulting in a danger to human health and the environment. The scheelite-type compounds have been investigated owing to their interesting photocatalytic properties in water purification trough the removal of different organic and inorganic pollutants. In this article a method solid state for doping of *bismuth(III) in* systems $Sr_{1-3x}Bi_{2x}\Phi_{x}MoO_{4}$ with *(*0 ≤ x ≤ 0.225) were obtained and, likewise its pelletizing process. Subsequently, these new materials were spectroscopically characterized with photocatalytic properties and finally is describe its development as oxidant against Rhodamine B. This work can be used for the synthesis of new Bi-doped strontium molybdates, which the best photochemical properties are chosen and, in turn, it is experimentally shown how can favor its absorption in the visible region. These electronic properties can be used in near studies, for to compressive the role of bismuth(III) in sheelite as photocatalyst and, to implement its use in the degradation of persistent pollutants that affect the world's water resources.

• The doping of bismuth(III) for systems $Sr_{1-3x}Bi_{2x}\Phi_{x}MoO_{4}$ modified the GAP absorption and this catalytic properties using this new solid state method.
• The degradation of Rhodamine B for systems $Sr_{1-3x}Bi_{2x}\Phi_{x}MoO_{4}$ as case study using of this methodology allows multiple applications associated with climate change such as: the degradation of emerging pollutants and the sensitization of semiconductors with solar claims.
• The role of bismuth(III) in these systems can be harnessed to design similar materials with photocatalytic properties.

## Graphical abstract

**Figure:**
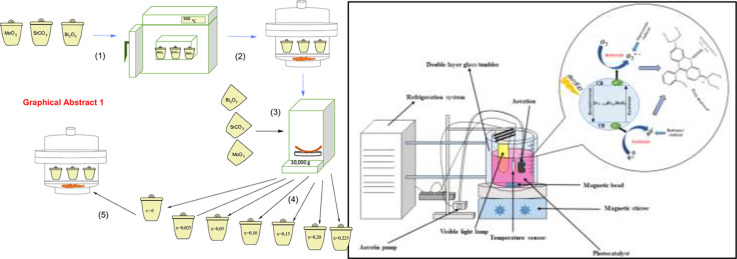

Specifications table

**Table:** 

Subject area:	Materials Science
More specific subject area:	*New functional materials*
Name of your method:	*Solid state reactions of new Bi-doped strontium molybdate synthesized as photocatalytic precursors for Rhodamine B degradation*
Name and reference of original method:	[1,2]
Resource availability:	*NA*

## Introduction

The scheelite-type compounds have been investigated owing to their interesting photocatalytic properties in water purification trough the removal of different organic and inorganic pollutants. These compounds are mainly semiconductors ABO_4_ (A = Sr, Pb; B = W, Mo) prepared by co-precipitation method generally. However, the pelletizing differs from other agglomeration techniques in that the powdered ore is first formed into a pellet or ball, which is then dried and hardened in a separate step, usually by heating. The pellets are then dried, preheated, and finally heated to a temperature below the melting point of iron oxides, to achieve strength. The fired pellets are typically strengthened by recrystallization across the particle grain boundaries. The ethanol plays an important role for powders $Sr_{1-3x}Bi_{2x}\Phi_{x}MoO_{4}$, favoring the sufficiently fine particle size distribution, a significantly humidity apport and a character as binder. This conditions at high temperatures stabilized the tetragonal structure for scheelites. These contains 8-coordinate, cations and (BO_4_)_n_^−^ tetrahedral anions which can be described within the space group I41/a. The A and B positions of cations can be occupied by ions with a charge from ^1+^ to ^4+^ and from ^3+^ to ^7+^ respectively. The scheelite-type materials may increase the valence band maximum to higher energy levels relative to the redox potentials of adsorbed molecules. However, on account of their wide bandgaps (*E_g_*= 4 - 5 eV), high energy- consuming ultraviolet radiation must be applied to studies relationship with the efficiency of photocatalytic processes. Therefore, its capacity to act as photocatalyst can be enhanced using bismuth(III) in the visible region. The doping in previously works showed changes in the dielectric properties by addition of this metal ion. The pelletizing method enhance the effective value of the permittivity increasing with the bismuth concentration in the solid solution. These electronic properties can be used in near studies, for to compressive the role of bismuth(III) in sheelite as photocatalyst.

On the other hand, also is showed as the effects on Eg, oxygen vacancies and reactive oxygen species can be explored thorough of a degradation methodology. For Rhodamine B was used this degradation method considering substation stoichiometric for $Sr_{1-3x}Bi_{2x}\Phi_{x}MoO_{4}$ with bismuth(III) and parameters such as time, concentration and exposition light [[3], [4], [5], [6], [7], [8], [9], [10], [11], [12], [1], [2], [13]].

In present work doping of SrMoO_4_ by bismuth was carried out by formation of cation vacancies and Bi:SrMoO_4_ ceramic were prepared from powders $Sr_{1-3x}Bi_{2x}\Phi_{x}MoO_{4}$ of complex oxides, using pelletizing method. Likewise, the electronic properties in solid state with structural parameters obtained of X- Ray diffraction patterns, particle size distribution function and IR spectra were correlated, for to understand the consequence in apply this solid method synthesis. Finally, the method degradation is described for Rhodamine B, using a new metal oxide synthesized emphasized parameters as concentration, time, reuse and light wavelength used.

## Materials and methods

### Materials

SrCO_3_ (99.5%), Bi_2_O_3_ (99.9%) and MoO_3_ (99.0%) as starting materials are obtained of sigma- Aldrich.

### Solid -state synthesis for $Sr_{1-3x}Bi_{2x}\Phi_{x}MoO_{4}$ compounds

**Fig. 1:**
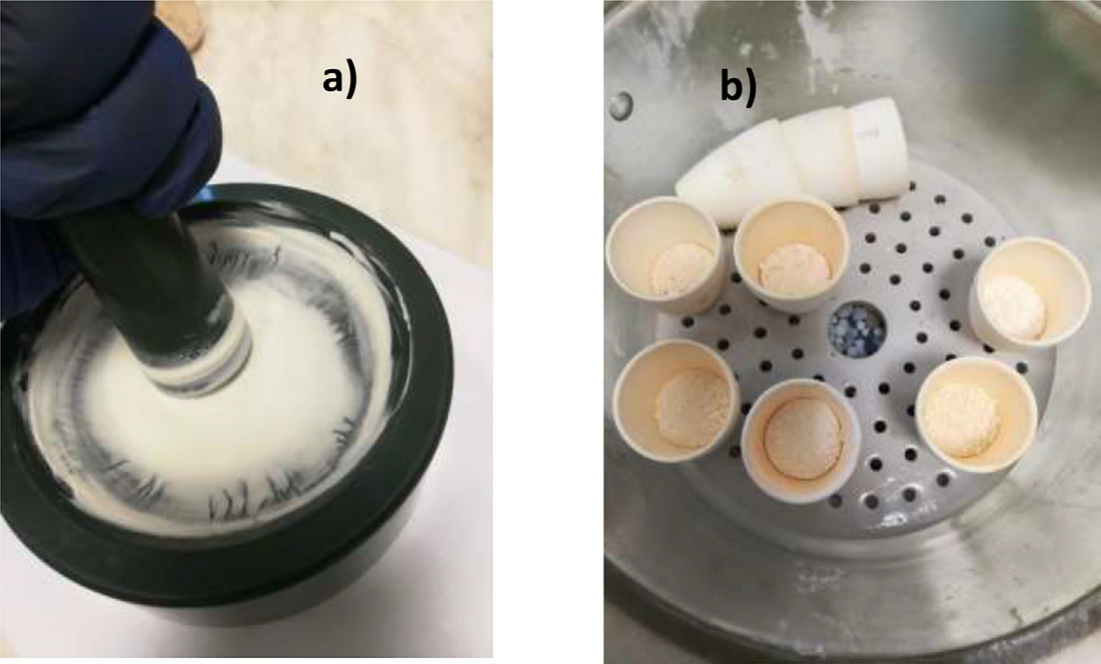
a) Grinding process for the sample *x* = 0.1 using ethanol as dispersant. b) Samples after the grinding process in the desiccator.

a. The synthesis was started with the crucible containing the sample labeled *X* = 0. This product was placed in a mortar to favors the reaction in the solid- state; the ethanol as dispersant, was used. The ethanol must be added and in simultaneous form a pestle paste is obtained.
b. The grinding process should be slow, and the force applied from the pestle to the sample in the mortar should be monitored, as to avoid loss of the sample by splashing.
c. At the end of the grinding process, the sample has the appearance of the powder and it is dried.
d. For the finalization of pelletizing process, the fine power at temperature of 500 °C for 8 h, was carried.
e. The sample is removed from the muffle furnace, cooled to room temperature and taken to the desiccator.
f. The process described from points a-c is repeated, for re- pelletizing at 600 °C, 650 °C and 700 °C for 8 h in each heating cycle.
g. The process described above is considering a stoichiometric relationship of x=0.025,0.05,0.10,0.15,0.20and0.225. Fig. 1.

### Spectroscopic characterization of $Sr_{1-3x}Bi_{2x}\Phi_{x}MoO_{4}$ materials

Band gap energies by the Kubelka-Munk method were calculated. X- Ray diffraction patterns for $Sr_{1-3x}Bi_{2x}\emptyset_{x}MoO_{4}$ in the range 0,025 ≤ *x* < 0,225, were carried. These patterns show all peaks at low angles include value for 10–25º 2θ, which were previously attributed to a secondary phase of unknown composition for x=0.20 and x=0.225. The particle size distribution function for oxide materials in suspensions by dynamic light scattering, were estimated. SALD-7101 Shimadzu particle size analyzer, was used with an arrangement of photosensors with a wide angle of coverage (60°), which it allows to improve the resolution in the range of submicron.

### Methodology for photocatalytic application with Rhodamine B

The photocatalytic activity of Sr_1−3x_ Bi_2x_Ф_x_MoO_4_ (0 ≤ *x* ≤ 0,025) system by the degradation of Rhodamine B in water solution (350 W lamp HPL-N (Philips), was evaluated.

a. The lamp emits to 365 nm with an energy of 4 eV.
b. An amount of 0.2 g of substrate to 200 mL for RhB solution (10 mg/L) in a double layer glass tumbler, was added.
c. Before illumination with the lamp, the suspensions were magnetically stirred for ~ 30 min by a magnetic stirrer (~ 400 rpm, to establishment an adsorption-desorption equilibrium between the photocatalyst and the RhB solution.
d. The total irradiation time was of 30 min, and tested in 1, 2, 5, 10, 15, 20 and 30 min. The samples were filtered and closed in test tubes to be analyzed using the Unico 2800 spectrophometer at 554 nm wavelength.
e. The absorbances and statistically analyzed to calculate the percentage of decolorization (D) of RhB, were recorded.
f. The temperature during the photocatalysis was 40,0 ± 1 ºC, controlled by a water recirculation system with a cooling system.
g. Excess oxygen was supplied during the suspension reaction using a corundum air diffuser aeration system.

## Results and discussion

### General chemical equation for the synthesis of complex oxide series using pelletizing method

(1) $$SrCO_{3}+Bi_{2}O_{3}+MoO_{3}\to Sr_{1-3x}Bi_{2x}\Phi_{x}MoO_{4}+CO_{2}$$

Eq. (1) shows the reaction through a grinding and pelletizing process, the series of complex oxides doped with Bi. The Φ represents the cation vacancies. Table 1 collects all the balanced chemical equations for each value of x replaced in the general formula $Sr_{1-3x}Bi_{2x}\Phi_{x}\text{Mo}O_{4}$.

**Table 1:** Balanced chemical equations of the complex oxides for each bismuth(III) doping.

x*-value*	*Balanced Chemical Equations*
x=0	$SrCO_{3}+MoO_{3}\to SrMoO_{4}+CO_{2}↑$
x=0.025	$0.925\;SrCO_{3}+0.025\;Bi_{2}O_{3}+MoO_{3}\to Sr_{0.925}Bi_{0.05}\Phi_{0.025}MoO_{4}+0.925\;CO_{2}↑$
x=0.05	$0.85\;SrCO_{3}+0.05\;Bi_{2}O_{3}+MoO_{3}\to Sr_{0.85}Bi_{0.1}\Phi_{0.05}MoO_{4}+0.85\;CO_{2}↑$
x=0.1	$0.7\;SrCO_{3}+0.1\;Bi_{2}O_{3}+MoO_{3}\to Sr_{0.7}Bi_{0.2}\Phi_{0.1}MoO_{4}+0.7\;CO_{2}↑$
x=0.15	$0.55\;SrCO_{3}+0.15\;Bi_{2}O_{3}+MoO_{3}\to Sr_{0.55}Bi_{0.3}\Phi_{0.15}MoO_{4}+0.55\;CO_{2}↑$
x=0.20	$0.4\;SrCO_{3}+0.2\;Bi_{2}O_{3}+MoO_{3}\to Sr_{0.4}Bi_{0.4}\Phi_{0.2}MoO_{4}+0.4\;CO_{2}↑$
x=0.225	$0.325\;SrCO_{3}+0.225\;Bi_{2}O_{3}+MoO_{3}\to Sr_{0.325}Bi_{0.45}\Phi_{0.225}MoO_{4}+0.325\;CO_{2}↑$

**Fig. 2:**
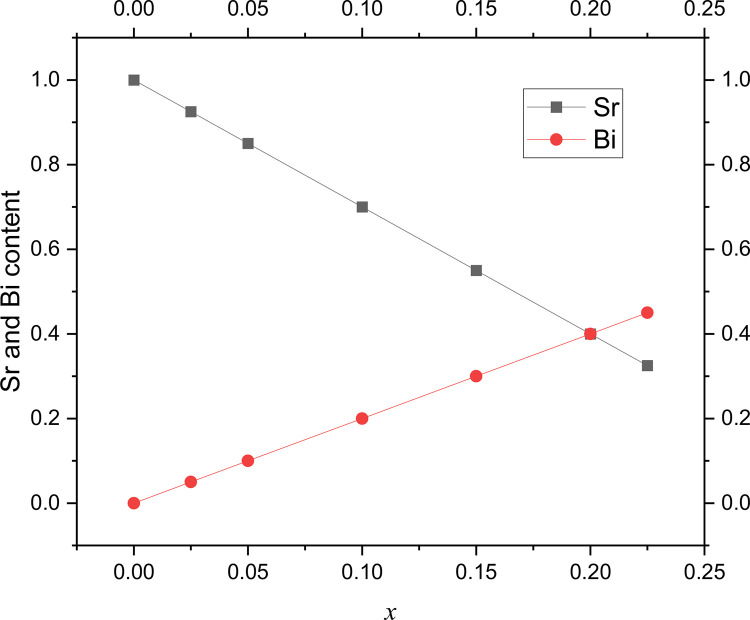
Relationships the Sr Vs Bi content of each complex oxide.

There is a relationship inversely proportional between Sr content Vs Bi, as is hoped, Fig. 2. Both linear relationships intercept for the sample with *x* = 0.20.

### Spectroscopic characterization of $Sr_{1-3x}Bi_{2x}\emptyset_{x}MoO_{4}$ materials

**Fig. 3:**
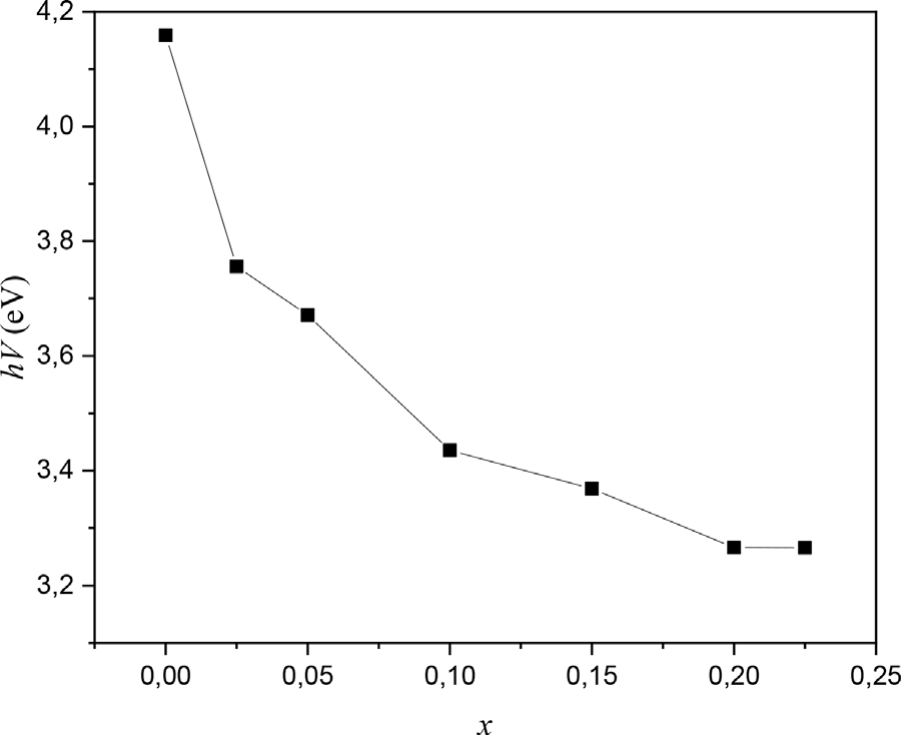
Band gap of the complex oxides obtained from the Diffuse reflectance spectrum and calculated by the Kubelka-Mulk method (0 ≤ *x* ≤ 0.025). It is observed that the GAP decreases with increasing Bi^3+^ content in the oxides.

Band gap energies by the Kubelka-Munk method were calculated. It is observed that the GAP decreases with increasing Bi^3+^ content in the complex oxides. Fig. 3.

**Fig 4:**
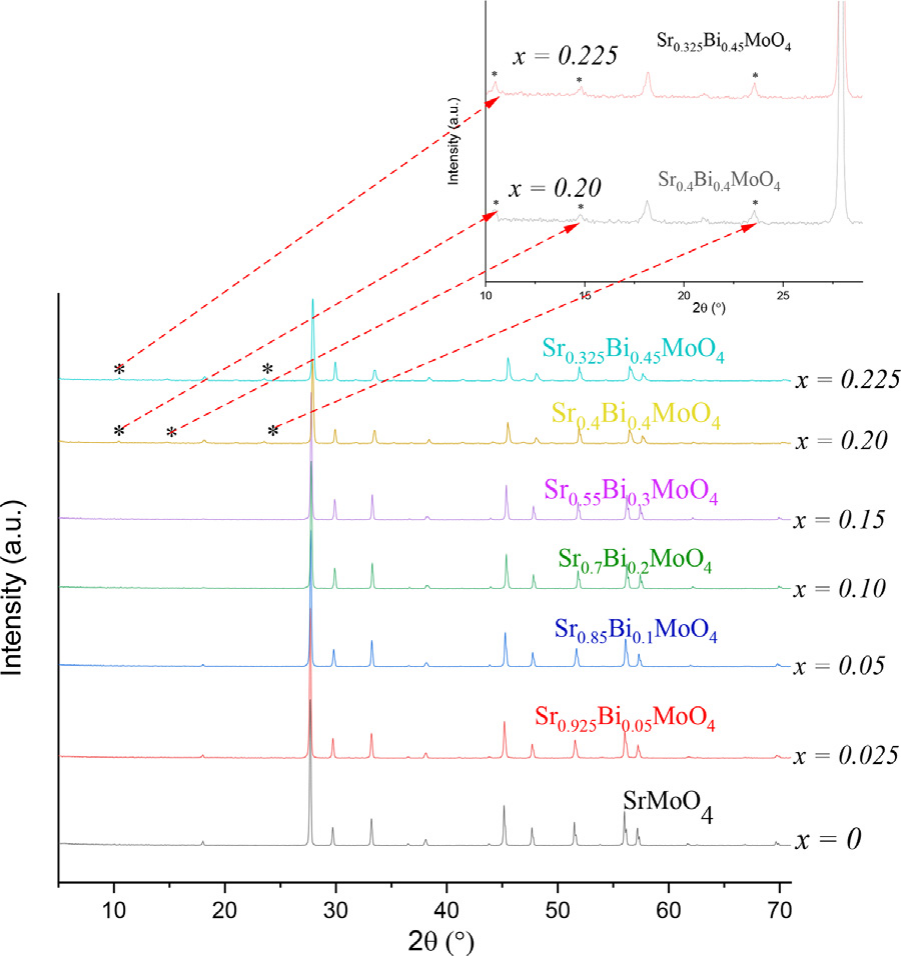
*XRD for*$Sr_{1-3x}Bi_{2x}\emptyset_{x}MoO_{4}$*.* Patterns in the range 0.025 ≤x≤0.225 show additional peaks (very weak for x=0.20 and x=0.225 compositions) at low angles 10–25º 2θ, which were previously attributed to a secondary phase of unknown composition.

**Fig. 5:**
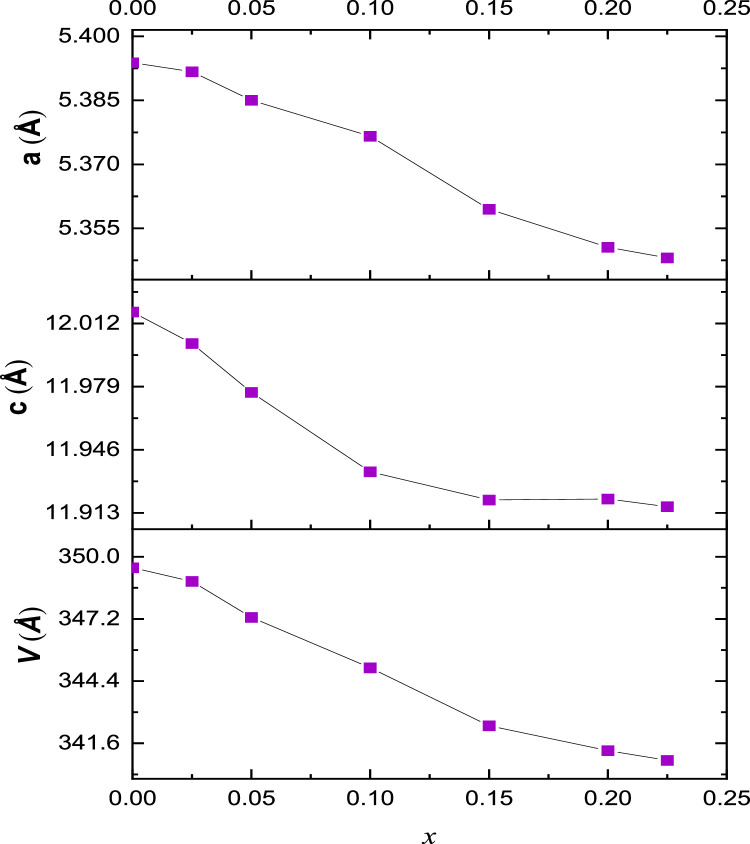
Composition dependence of unit cell parameters a, c and volume for *Sr_1−3x_Bi_2x_Ф_x_MoO_4_*.

X- Ray diffraction patterns for $Sr_{1-3x}Bi_{2x}\emptyset_{x}MoO_{4}$ in the range 0,025 ≤ *x* < 0,225 showed the substitution in target cell. The cell parameters for each complex oxide were calculated observing that it decreased as the bismuth content decreased. This behavior is attributed to the substitution of Sr^2+^ by larger Bi^3+^ cations with ionic radii of 1.13 Å and 1.17 Å, affecting the volume and a and b parameters in cell crystalline. Figs. 4 and 5.

**Fig 6:**
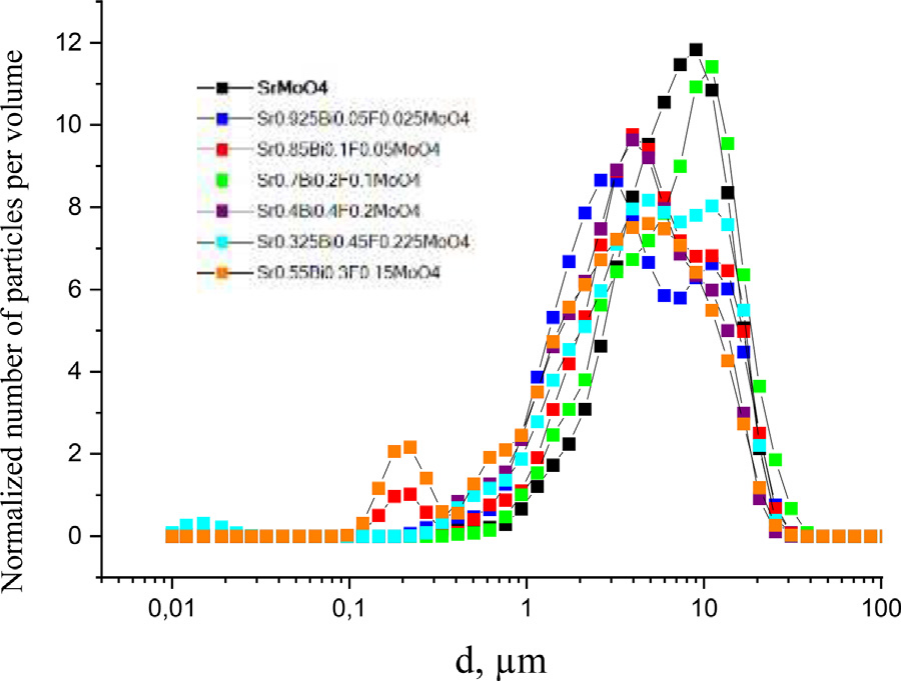
The variation of the diameter of the particles by volume for $Sr_{1-3x}Bi_{2x}\emptyset_{x}MoO_{4}$ in the range 0.025 ≤ *x* < 0.225.

The Table 2 showed the maximum and minimum values for the diameter particles and the percentages of the total number of particles. Finally, the variation of the diameter of the particles by volume, Fig. 6.

**Table 2:** Maximum and minimum values of the diameter of the particles and the percentages of the total number of particles that have the same diameter.

№	Sample	10%D, μm	50%D, μm	90%D, μm	d***, μm	d_min_, μm	d_max_, μm
1	SrMoO_4_	2.1	6.0	12.97	8.1	0.3	31.1
2	Sr_0.925_Bi_0.05_F_0.025_MoO_4_	1.2	3.7	13.4	2.6	0.2	38.2
3	Sr_0.85_Bi_0.1_F_0.05_MoO_4_	1.2	4.0	12.3	3.6	0.1	31.1
4	Sr_0.7_Bi_0.2_F_0.1_MoO_4_	1.9	6.6	14.9	10.0	0.4	38.2
5	Sr_0.55_Bi_0.3_F_0.15_MoO_4_	0.5	3.3	10.6	4.4	0.1	31.1
6	Sr_0.4_Bi_0.4_F_0.2_MoO_4_	1.0	3.7	11.0	3.6	0.3	25.3
7	Sr_0.325_Bi_0.45_F_0.225_MoO_4_	1.2	5.1	15.5	4.4	0.1	34.4

*dmin is the minimum particle size (there are no particles with a smaller diameter); dmax is the maximum particle size (there are no particles with a larger diameter); 10%D - 10% of the total number of particles have a given diameter; 50%D - 50% of the total number of particles have a given diameter; 90%D - 90% of the total particles have this diameter*.

**Fig 7:**
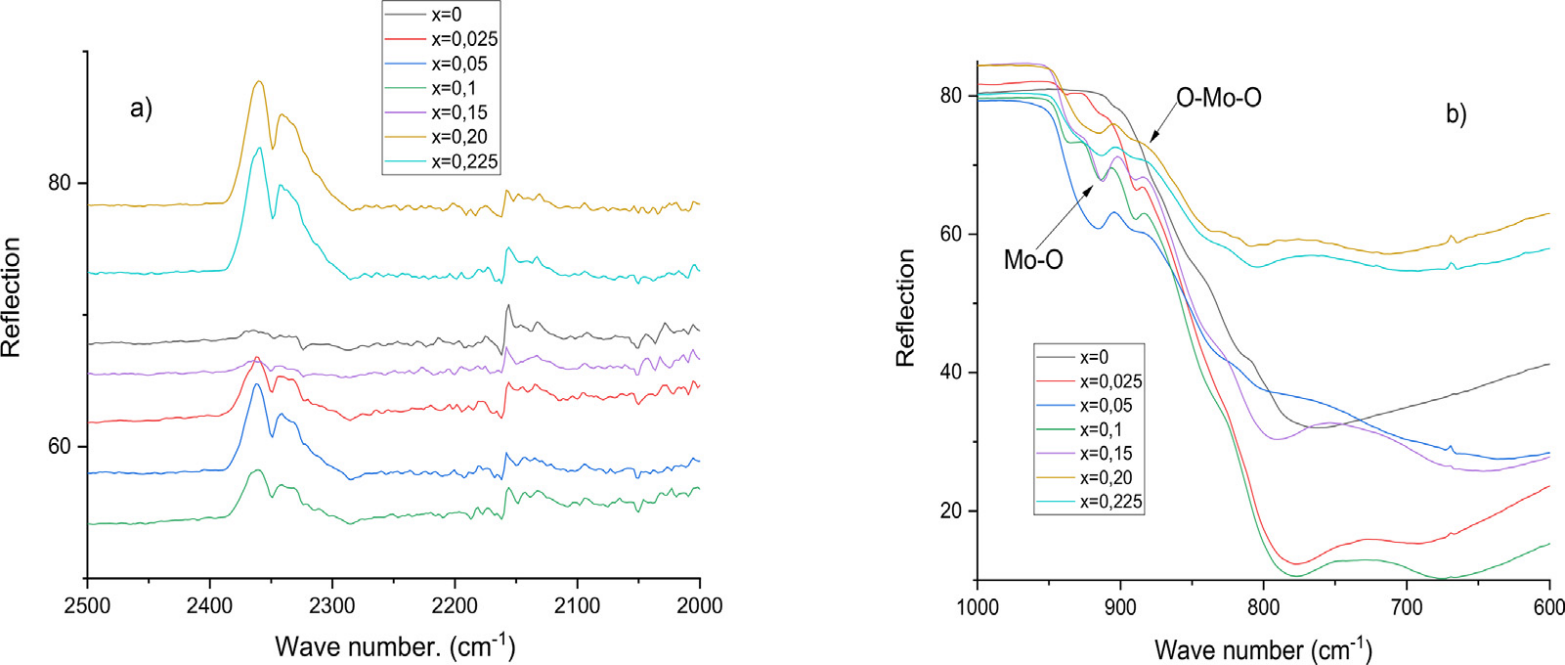
**a).** IR spectra for $Sr_{1-3x}Bi_{2x}\emptyset_{x}MoO_{4}$ for 0.025 ≤x≤0.225, b). Mo-O and O-Mo-O IR region.

On the other hand, the next figure shows the IR spectra. The peaks at 1345 cm^–1^ correspond with the frequency (C—O) for vibrational modes in IR spectroscopy indicates the characteristic deformation in the bond Sr – O, Mo – O, and Mo – O – Mo bridges, as expected. Fig. 7.

### Method validation for photocatalytic application with Rhodamine B

**Fig. 8:**
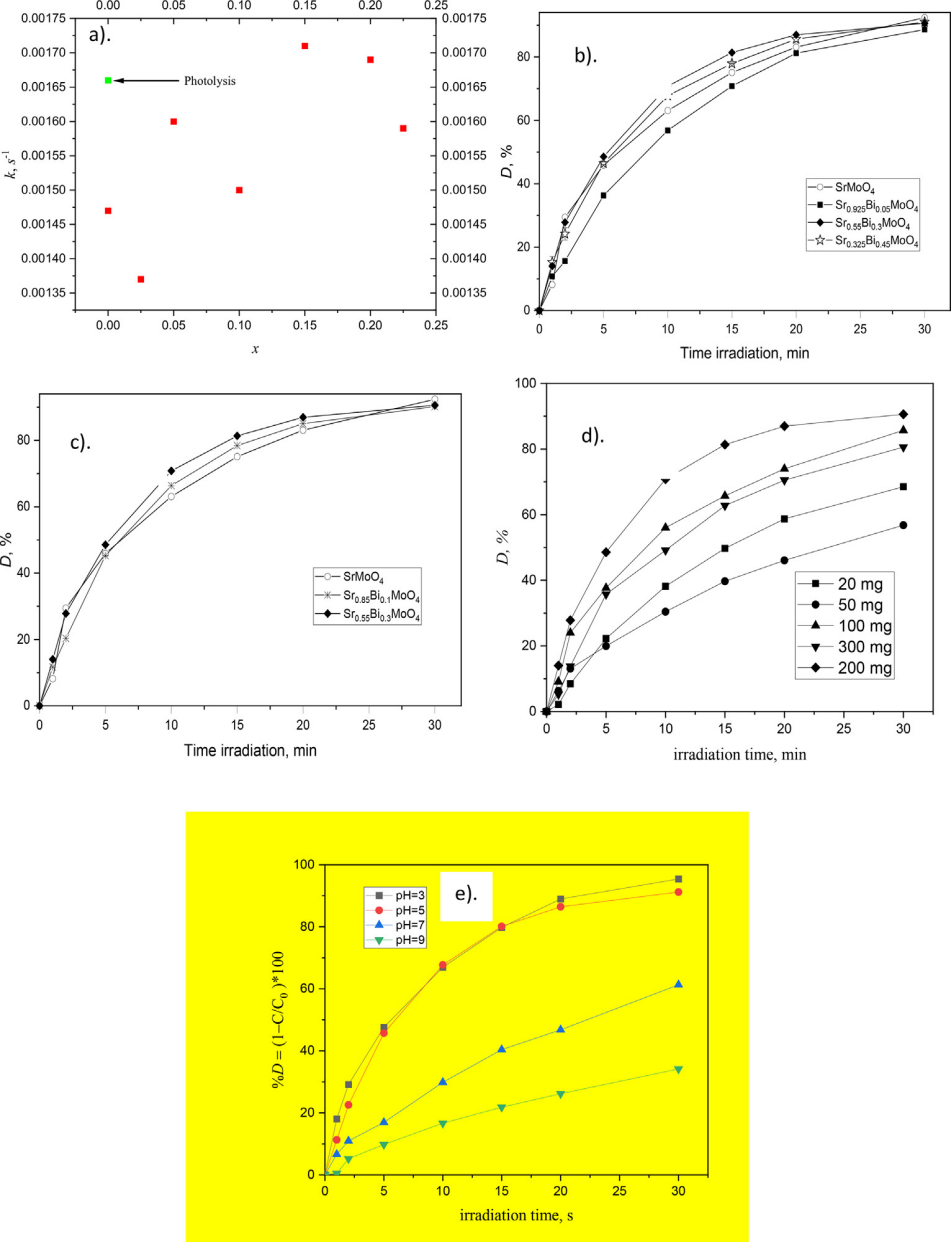
a). Reaction rate constants of each complex oxide against RhB, photolysis is shown, b) and c) RhB degradation for $Sr_{1-3x}Bi_{2x}\emptyset_{x}MoO_{4}$ (*x* = 0.05- 0.25), d). Dosses catalyst, e).pH effect.

The photocatalytic activity of the $Sr_{1-3x}Bi_{2x}\Phi_{x}MoO_{4}$ system was investigated for oxidation of Rhodamine B. showed for all compositions a pseudo-first-order kinetic model, with the concentration of RhB changing according to the rate law, Eq. (2).

(2) $$C=C_{0}e^{-kt}$$

where C is the concentration of RhB at time *t*, $C_{0}$ is the initial concentration of RhB and *k* is the reaction rate. The sample with x=0.15 presented a best adjust when oxidizing RhB in an aqueous medium at pH 3.0. In the Fig. 8a is showed that for a stoichiometric *X* = 0.15 and 0.20, the degradation is higher with relationship to Photolysis. However, for stoichiometric *X* = 0.05, 0.10 and 0.25 the degradation does not enhance. This behavior is correlated with minor value E_g_ and minor particle size. In studies on photocatalysis processes, comparison of the results of the photolysis, adsorption and photocatalytic mechanisms is important. Thus, the mentioned processes were respectively differentiated in the experiments in the presence of UV radiation, but without a catalyst to assay the photolysis process and in dark condition to evaluate the adsorption process. Based on the 4.1%, 20.4%, and 92.7% removal efficiency for adsorption, photolysis, and photocatalysis, respectively, RhB removal by $Sr_{1-3x}Bi_{2x}\emptyset_{x}MoO_{4}$ *X* = 0.15 occurs mainly through photocatalytic degradation. The effects of initial $Sr_{1-3x}Bi_{2x}\emptyset_{x}MoO_{4}$ (*x* = 0.05- 0.25), pH (3, 5, 7, 9), dosses catalyst (20, 50, 100, 200, 300 mg), and UV-C irradiation time (0, 5, 10, 15, 20, 25 and 30 min) were optimized in a batch photoreactor, using RhB with 10 mg/L, **figure b, c, d and e.** The photoreactor was a rectangular cubic shape, made from Plexiglas, and had internal dimensions of 50 cm (length), 25 cm (width), and 2.5 cm (height), an applicable volume of 300 mL, and three UV lamps (low pressure, 6 W, Philips) that were placed on top of the reactor. The samples were taken at the definite interval of times during the irradiation; after the separation of $Sr_{1-3x}Bi_{2x}\emptyset_{x}MoO_{4}$ by filtration and the samples were analyzed by UV- VIS spectrophotometer.

The photocatalytic performance of $Sr_{1-3x}Bi_{2x}\emptyset_{x}MoO_{4}$ for degradation of RhB was compared in the optima conditions: pH= 3.0, irradiation time of 30 min, RhB concentration of 10 mg/L, and photocatalyst loading of 0.2 g. The RhB removal efficiency with the photocatalysts for $Sr_{1-3x}Bi_{2x}\emptyset_{x}MoO_{4}$was 92.7% with *x* = 0.15.

**Fig. 9:**
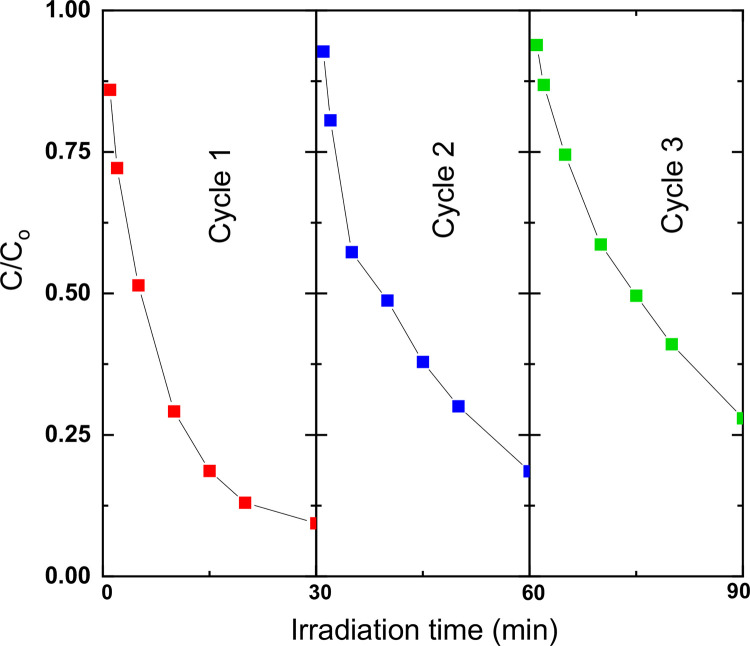
First, second and three reuse cycles for RhB degradation in the system *x* = 0.15.

Finally, the reuse cycle was evaluating for the *X* = 0.15 system founding possible adsorption effects between material and RhB, which would less the remotion low these conditions for cycle 2 and 3. Fig. 9.

## Conclusions

A cation vacancy solid solution is formed in the system Sr_1−3x_Bi_2x_*Ф*_x_MoO_4_ for the compositional range 0 ≤ *x* ≤ 0.225, using pelletizing method. Phases in the range 0.025 ≤ *x* ≤ 0.15 show a tetragonal scheelite structure favored with this synthesis. The photocatalytic activity of Sr_1−3x_Bi_2x_*Ф*_x_MoO_4_ compositions is similar to behavior with SrMoO_4_ and photolysis for RhB for 0.025 ≤ *x* ≤ 0.10, using this degradation method. However, with *x* = 0.15 and 0.20 the degradation RhB (10 mg/L) at pH acid showed an 92.7% of remotion with 30 min treatment and 200 mg of Catalyst. These results are interesting, due a that is possible to explore to remotion for RhB with this material, using these synthesis and degradation conditions. The cationic vacancies can be enhanced for other authors and to use this compounds family in similar molecules as Rh considering the solid state method for synthesis and after this RhB degradation for systems with *X* = 0.15 and 0.20 stichometry.

## CRediT author statement

**Jorge Acosta-Vergara:** Conceptualization, Methodology, Software, Writing- Original draft preparation, visualization and Investigation. **Yenny Ávila- Torres**: Conceptualization, Methodology, Validity tests, Data curation, Writing- Original draft preparation, visualization and Investigation.  **Ricardo Torres- Palma:** visualization and Investigation.

## Declaration of Competing Interest

The authors declare that they have no known competing financial interests or personal relationships that could have appeared to influence the work reported in this paper.
